# Morphological redescription and taxonomic reassignment of *Deraiophoronema evansi* (Lewis, 1882) Romanovitch 1916 n. comb. (syn: *Dipetalonema evansi*) (Spirurida: Onchocercidae) from camels

**DOI:** 10.1186/s13071-025-07019-z

**Published:** 2025-10-10

**Authors:** Coralie Martin, Jules Rodrigues, Frédéric Fercoq, Nathaly Lhermitte-Vallarino, Alireza Sazmand, Daisuke Kimura, Shigehiko Uni

**Affiliations:** 1 UMR7245 MCAM, Museum National d’Histoire Naturelle, CNRS, CP52, 61 Rue Buffon, 75005 Paris, France; 2 Department of Pathobiology, Faculty of Veterinary Medicine, Bu-Ali Sina University, Hamedan, 6517658978 Iran; 3 Department of Health, Sports and Nutrition, Faculty of Health and Welfare Studies, Kobe Women’s University, Kobe, 650-0046 Japan

**Keywords:** Filaria, Nematode, Camels, Setariinae, Multi-locus sequence analyses, Phylogeny, Taxonomy

## Abstract

**Background:**

Filarioses are common nematode infections in camels (*Camelus* spp.). The most significant disease is caused by *Deraiophoronema evansi*, which impacts camel reproductive function, working ability, and productivity. The taxonomy of this onchocercid is equivocal, and its phylogenetic relationships within Onchocercidae are ambiguous.

**Methods:**

We analyzed *D. evansi* specimens from camels and examined their morphology. For comparative material, we analyzed fresh specimens from camels in Iran and specimens from Egypt deposited in the Muséum National d’Histoire Naturelle (MNHN) collections. Multi-locus sequence analyses based on seven genes (two mitochondrial genes *cox1* and *12S* ribosomal DNA (rDNA) and five nuclear genes *18S* rDNA, *28S* rDNA, *MyoHC*, *rbp1*, and *hsp70*) of these filarioids and six genes of *Wolbachia* (*16S* rDNA, *ftsZ*, *dnaA*, *coxA*, *fbpA*, and *gatB*) were analyzed.

**Results:**

*Deraiophoronema evansi* (Lewis, 1882) Romanovitch 1916 *combinatio nova* (n. comb.)(syn: *Dipetalonema evansi*) was described on the basis of morphological characteristics and its genetic divergence from congeners. Molecular characteristics of the new species revealed its close evolutionary relationship with *Setaria* sp. *Wolbachia* endosymbiont was not present in *D. evansi*.

**Conclusions:**

We provide new molecular and morphological data on *D. evansi*, increasing the number of valid genera of Setariinae to four and setting up the taxonomic information regarding this species of veterinary importance.

**Graphical abstract:**

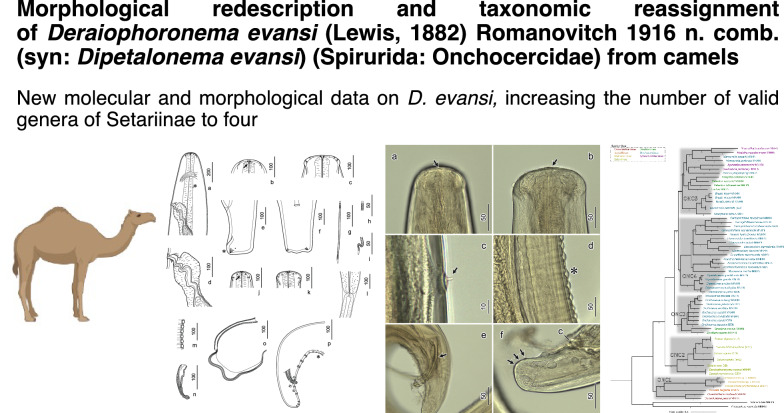

**Supplementary Information:**

The online version contains supplementary material available at 10.1186/s13071-025-07019-z.

## Background

Filarial parasites (Nematoda: Onchocercidae) are found in a broad spectrum of ungulate hosts [1]. Among them, species of the *Onchocerca* genus and the filarial *Deraiophoronema evansi* (Lewis, 1882) Romanovitch 1916 *combinatio nova* (n. comb.) (syn: *Dipetalonema evansi*) (Spirurida: Onchocercidae) [2] are widespread in Old World camels, i.e., *Camelus bactrianus* and *C. dromedarius* [3–6]. This nematode parasitizes the pulmonary and internal spermatic arteries, lymph nodes, and lymph vessels of camels and is transmitted by mosquitoes [7–9]. It has been described in many countries from Northern Africa and the Sahel, the Middle East, and Central/South/East Asia, and it has also been introduced to Australia [7–10]. Clinical manifestations include weakness, loss of appetite, orchitis, aneurysms in the spermatic cord, arteriosclerosis, and heart failure, leading to significant economic losses [7–9].

This species is a taxonomic challenge. Since its original description as *Filaria evansi* Lewis 1882 [2], it was renamed *Acanthocheilonema evansi* (Lewis, 1882) Baylis et Daubney, 1923 [11, 12], then *Setaria evansi* (Lewis, 1882) Baylis et Daubney, 1926 [13], and finally *Dipetalonema evansi* (Lewis, 1882) Yorke et Maplestone, 1926 [14–18] (Table 1). This filarial species was also reported as *Microfilaria sanguinicolae* Cazalbou, 1916 [19], *Microfilaria camelensis* Balfour, 1911 [20], *Filaria haematica cameli* Pricolo, 1913 [21], and *Deraiophoronema cameli* Romanovitch, 1916 [22]. Later, in 1952, it was established that the morphological characteristics of *Dipetalonema evansi* were considerably different from those of the genus *Dipetalonema* [23]. Then in 1953, it was proposed to put in synonymy *Dipetalonema evansi* with *Deraiophoronema cameli* Romanovitch, 1916, and to finally name the species *Deraiophoronema evansi* (Lewis, 1882) (syn: *Deraiophoronema cameli* Romanovitch, 1916; *Dipetalonema evansi*) [24].

**Table 1:** History of taxonomic changes in *Deraiophoronema evansi*

Author(s)	Year	Parasite name	Host	Country	Available material		Measures
Lewis	1882	*Filaria evansi*	Camel	Pakistan	Not specified	No	Yes
Romanovitch	1916	*Deraiophoronema cameli*	*Camelus bactrianus*	Kyrgyzstan	One full male, one anterior part of male, pieces of female	No	Yes
Baylis and Daubney	1923	*Acanthocheilonema evansi*	Camel	Pakistan	One female, one posterior part of male	Yes	Yes
Boulenger	1924	*Acanthocheilonema evansi*	Camel	Pakistan	Females and males	Yes	Yes^*^
Baylis and Daubney	1926	*Setaria evansi*				No	No
York and Mapplestone	1926	*Dipetalonema evansi*				No	No
Boulenger	1928	*Dipetalonema evansi*				No	No
Baylis	1939	*Dipetalonema evansi*				No	Yes^**^
Nagaty	1947	*Dipetalonema evansi*	*Camelus dromedarius*	Egypt	Females and males	Yes	Yes
Chabaud and Choquet	1953	*Deraiophoronema evansi*				No	No
Yeh	1957	*Deraiophoronema evansi*				No	No
Sonin	1975	*Dipetalonema evansi*				No	Yes^***^

Each column shows the author, the year, and the given name of the parasite. In addition, the host, the country of sampling, and the available material are provided. The presence of figures and/or measurements is mentioned

^*^ Same material as Baylis and Daubney (1923)

^**^ Based on drawings from Boulenger (1924)

^***^ Based on drawings from Nagaty (1947)

The genus *Deraiophoronema* was validated in 1957 with three species [25]. However, in 1976, two out of the three *Deraiophoronema* species were transferred to the genus *Bostrichodera* [26], and later in 1983, one of these species was transferred to the genus *Chabfilaria* [27]. Currently, the *Deraiophoronema* genus is monotypic with only *D. evansi*. However, since *Der. evansi* was rarely cited as a synonym of *Dip. evansi* [28–30], the validity of this genus has never been questioned. In addition, the reference to *Dip. evansi* by Sonin [18] was based on an earlier illustration by Nagaty [17], which likely contributed to confusion regarding the continuity of the name *Der. evansi.*

The morphological information is sparse, and it is essential to establish a robust morphological description of this species to reassign it. In addition, although some measurements were given by Lewis [2] and Romanovitch [22], no illustrations were shown, and no type material was defined.

Here we provide a new morphological analysis of specimens from two geographical origins, revealing new characteristics. Using a multi-gene dataset analysis based on seven loci (two mitochondrial and five nuclear genes) that we previously set up [31], we also performed a molecular analysis of this species. Altogether, the genus *Deraiophoronema* was valid and monotypic, and *Deraiophoronema evansi* was placed in the Setariinae clade at the basis of the genus *Setaria*.

## Methods

### Collection of hosts and parasites

Specimens from Iran were collected from the pulmonary artery of a slaughtered one-humped dromedary (*Camelus dromedarius*) in southeastern Iran in 2014. Specimens were also obtained from *C. dromedarius* in 1932 at the slaughterhouse in Cairo, Egypt. Originally, the specimens were deposited in the Parasite Collection of the Medical University of Paris V. Later, the collection was given to the parasite collection of the Muséum National d’Histoire Naturelle (MNHN), and the whole collection was registered under the no. BB459.

### Morphological methods

Adult worms (two males and five females from Egypt and three females from Iran) kept in 70% ethanol were used for morphological examination. Specimens were cleared in lactophenol (R & M Chemicals, Essex, UK) and drawn under a compound microscope equipped with a camera lucida (Olympus U-DA, Olympus, Tokyo, Japan).

For each worm, we recorded body length and width, distance between the anterior extremity and nerve ring, distance between the anterior extremity and vulva, and length of esophagus, spicules, and tail. We also recorded the length and width of microfilariae taken from the uteri of fixed adult females.

### Molecular analysis of filarial nematodes

The fragments of two females from Iran (ID no. MNHN-IN-112YT) were used to extract their DNA with the QIAamp DNA Micro Kit (Qiagen, Hilden, Germany). Samples were incubated at 56 °C with proteinase K for 4 h. Polymerase chain reaction (PCR) DNA amplification targeted partial sequences of seven genes, including two mitochondrial genes, i.e., *12S* ribosomal DNA (rDNA) (ca. 450 base pairs (bp)) and *cox1* (ca. 600 bp), and five nuclear genes, i.e., *18S* rDNA (ca. 740 bp), *28S* rDNA (ca. 900 bp), *MyoHC* (ca. 785 bp), *rbp1* (ca. 640 bp), and *hsp70* (ca. 610 bp). The final volumes of the PCR reactions were 20 µL [31, 32]. Samples were sequenced using the Sanger method by Eurofins Scientific and cleaned using Chromas (http://www.technelysium.com.au/chromas.html).

### Phylogenetic analyses

The dataset used to infer the Onchocercidae phylogenies was a combination of sequences from the present study and from literature (Additional File 1: Supplementary Table S1). For each gene, the multiple sequence alignments (MSAs) were generated using SATe version 2.2.7 [33], and then the MSAs were concatenated. The analyses comprise 62 taxa representing 58 species and 25 genera. A phylogeny based on the maximum likelihood (ML) was implemented. For each gene, the best-fitting substitution model was determined using the corrected version of the Akaike information criterion (AICc) in JModelTest version 2.1.10 analyses [34]. The TVM + I + G was the best fit for *12S* rDNA; TPM2 + I + G for *18S* rDNA; TVMef + I + G for *28S* rDNA; TIM1 + I + G for *cox1*; TPM1uf + I + G for *hsp70*; TrN + I + G for *myoHC*; and TPM3uf + G for *rbp1*. To root the trees, two species were included as the outgroups: *Filaria latala* Chabaud and Mohammad, 1989 (Spirurida: Filariidae) and *Protospirura muricola* Gedoelst, 1916 (Spirurida: Spiruridae). The phylogenetic relationships of the Onchocercidae were inferred by the ML on the partitioned concatenated dataset. The program was executed by generating ten random start trees with 1000 bootstraps using RaxML-NG [35].

In addition, a Bayesian inference (BI) was used to infer their phylogenetic relationships with MrBayes 3 [34]. The global MSA was partitioned for each gene to estimate the weighted average of the best-fit evolution models in the GTR + I + G landscape according to the different genetic markers. Two runs were performed using 7 million steps with four chains, with tree sampling every 7000 generations; the first 25% of the trees were discarded as “burn-in,” and posterior probabilities were calculated from the remaining trees.

### *Wolbachia* molecular screening

We screened the specimens for *Wolbachia* symbionts by nested PCR covering six genes (*16S* rDNA, *ftsZ*, *dnaA*, *coxA*, *fbpA*, and *gatB*) as described previously [36].

## Results

### Redescription of *Deraiophoronema evansi* (Lewis, 1882) Romanovitch 1916 n. comb.

#### Taxonomic summary

Phylum: Nematoda Rudolphi, 1808

Class: Chromadorea Inglis, 1983

Order: Spirurida Railliet, 1914

Superfamily: Filarioidea Weinland, 1858

Family: Onchocercidae Leiper, 1911

Subfamily: Setariinae Yorke and Maplestone, 1926

Genus: *Deraiophoronema* Romanovitch, 1916

Species: *evansi* Lewis, 1882

Type material: Specimens from Egypt, neotype (female 1, MNHN-IN-110YT), neotype (male 1, MNHN-IN-110YT), and paraneotypes (MNHN-IN-111YT [females 2 to 5, male 2]), were deposited in the Muséum National d’Histoire Naturelle (MNHN) in the National Nematode Zooparasites Collection (Paris, France). Specimens from Iran: [MNHN-IN-112YT (3 females)] were deposited in the Muséum National d’Histoire Naturelle (MNHN) in the National Nematode Zooparasites Collection (Paris, France).

Representative DNA sequences: Nucleotide sequences of seven genes were submitted to the GenBank database under the accession nos. PP815116, PP815118, PP815119, PP812516, PP848833, PP848834, and PP815119.

#### Optical microscopy of present specimens

Measurements of a representative male and female of *D. evansi* (from Egypt) are presented first, followed by the range, including the representative specimens in parentheses and the mean in brackets. Measurements are in micrometers unless otherwise stated.

**Fig. 1:**
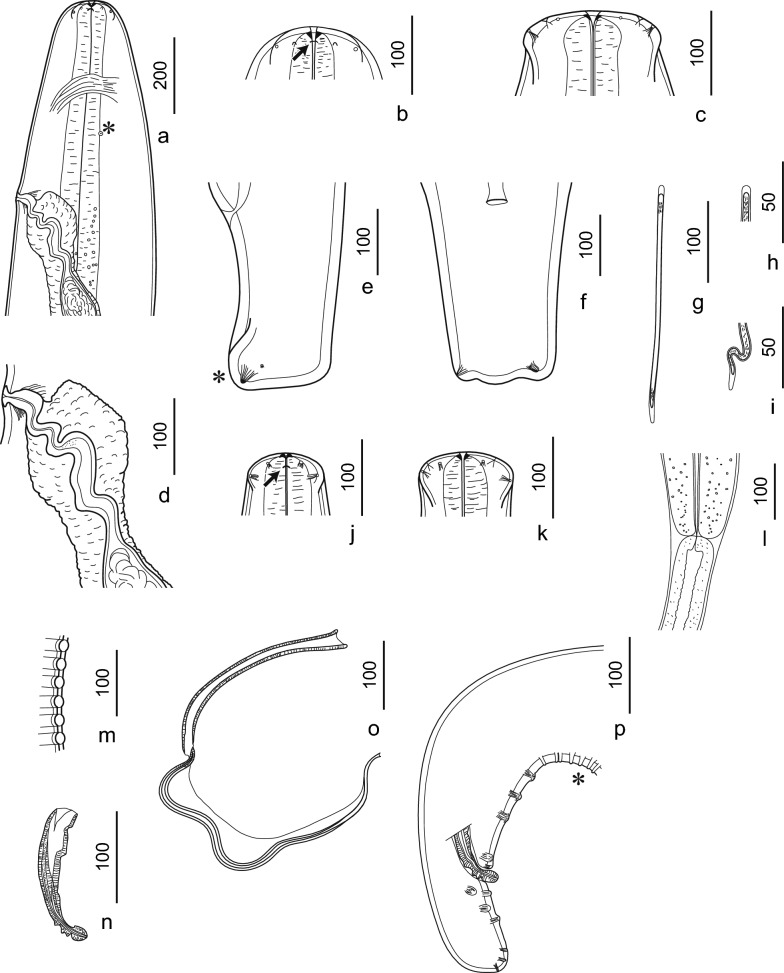
Line drawings of *Deraiophoronema evansi*. Females (**a**–**f**), microfilariae (**g**–**i**), and males (**j**–**p**). (**a**) Anterior end, left lateral view. Deirid (^*^). (**b**) Lateral view of anterior extremity, showing amphid (arrow). (**c**) Dorsoventral view of anterior extremity, showing rectangular formation stretched laterally. (**d**) Vagina, left lateral view. (**e**) Posterior end, lateral view. Large hump (^*^). (** f**) Posterior end, dorsoventral view. (**g**) Microfilaria with sheath. (**h**) Head with heath. (**i**) Tail tip with sheath. (**j**) Anterior extremity, lateral view, showing amphid (arrow). (**k**) Dorsoventral view of anterior extremity showing rectangular formation stretched laterally. (**l**) Esophago–intestinal junction. (**m**) Lateral view of area rugosa showing transverse bands. (**n**) Right spicule, lateral view. (**o**) Left spicule, lateral view. (**p**) Posterior end, right lateral view. Area rugosa (^*^). Scale bars are in micrometers

General description: Body elongated, cylindrical almost along entire length. Anterior end with cuticular rectangular formation stretched laterally in both sexes. Oral opening narrow, four labial papillae, and four cephalic papillae; two minute pore-like amphids lateral (Fig. 1b, c, j, k). Buccal capsule with pre-esophageal cuticular ring. Esophagus visibly divided into short muscular and longer glandular sections. Nerve ring encircling the muscular esophagus at the level of its anterior third. Deirids at mid-muscular esophagus.

**Fig. 2:**
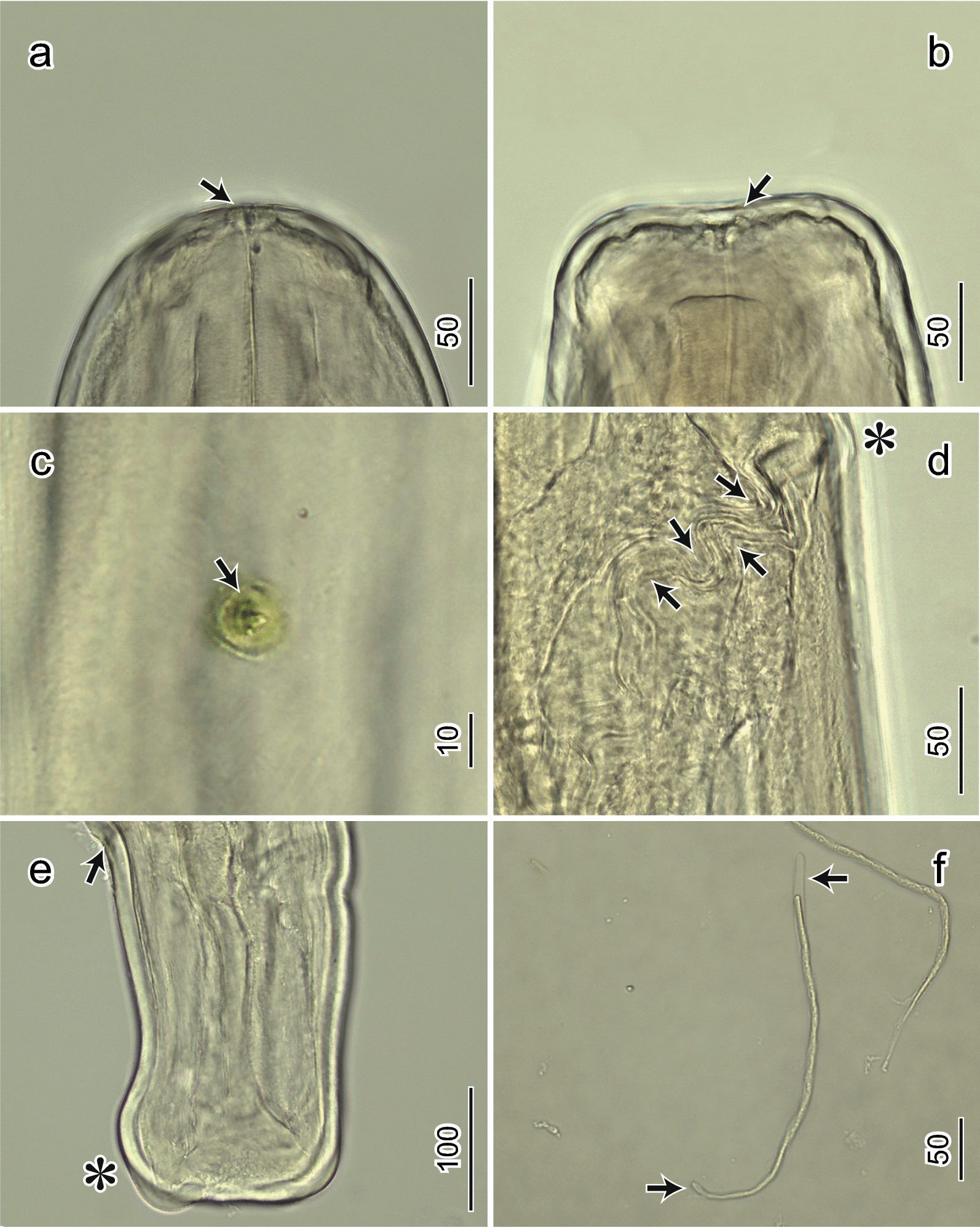
Light micrographs of *Deraiophoronema evansi*. Females (**a**–**e**) and microfilaria (**f**). (**a**) Lateral view of anterior end showing pre-esophageal cuticular ring (arrow). (**b**) Dorsoventral view of anterior end, showing rectangular formation stretched laterally. Pre-esophageal cuticular ring (arrow). (**c**) Deirid, en face view (arrow). (**d**) Right lateral view showing vulva opening (^*^) and globular vagina uterina and its bent lumen (arrows). (**e**) Lateral view of posterior end with anus (arrow) and large hump (^*^). (**f**) Microfilaria with sheath (arrows). Scale bars are in micrometers

Female: (*n* = 8) (Table 2; Figs. 1a–f, 2a–e). Body, 180 (165–185) [175] mm long; width at mid-body, 470 (470–770) [589]. Buccal capsule with pre-esophageal cuticular ring, 9 (5–11) [8.5] high and 27 (23–27) [25.2] wide (Fig. 1b, c). Muscular esophagus, 700 (700–960) [822] long. Glandular esophagus, 5.47 (5.47–7.05) [6.23] mm long. Total esophagus, 6.17 (6.17–8.4) [7.1] mm long. Nerve ring, 237.5 (200–238) [219] from the anterior end (Fig. 1a). Deirid, 470 (243–475) [349] from the anterior end (Figs. 1a, 2c). Vulva transversely split 820 (450–820) [630] from anterior end (Figs. 1a, 1d, 2d). Tail tapering, 250 (250–325) [289] long (Fig. 1e) with large hump at tip (Figs. 1e, 2e). Measurements are in micrometers unless otherwise stated.

**Table 2:** Comparative morphometric data from *Deraiophoronema evansi* females and congeners recorded from camels. Measurements are in micrometers unless otherwise stated

Characteristics	Present study, range								Lewis 1882	Baylis et Daubney 1923	Boulenger 1924	Nagaty 1947	Sonin 1975^*^
Country	Egypt	Egypt	Egypt	Egypt	Egypt	Iran	Iran	Iran	Pakistan	Pakistan	Pakistan	Egypt	Egypt
Examined material (no.)	Female (1)	Female (2)	Female (3)	Female (4)	Female (5)	Female (1)	Female (2)	Female (3)	Female	Female	Female	Female	Female
Body length (mm)	180	170	175	185	165				152–203	210	170–215	145–185	148–210
Body width	470	600	500	550	560	580	770	680	508–793	730	550–650	616–792	414–532
Pre-esophageal cuticular ring (height/diameter)	9					5	8.75	11.25					
	27					26.3	22.5	25					
Nerve ring from anterior end	237.5	200	220	222	225	212.5	209	225		204	200		180–200
Deirids from anterior end	470	475	475	400	275	350	350	242.5					
Vulva from anterior end	820	780	720	580	450	600	555	537.5	635	595	570–650	495–686	450–612
Muscular esophagus length	700					930	700	960			750–800	540–792	646–810
Glandular esophagus length (mm)	5.47						7.05	6.16			5.5–5.8	5.0–6.25	5.76–7.15
Junction of esophagus/intestine (mm)	6.17						7.75	7.12					
Tail length	250	250	325	300	325	255	320		254	238	250–300	279–440	216–324

Measurements are in micrometers unless otherwise stated

^*^Based on drawings from Nagaty 1947

**Fig. 3:**
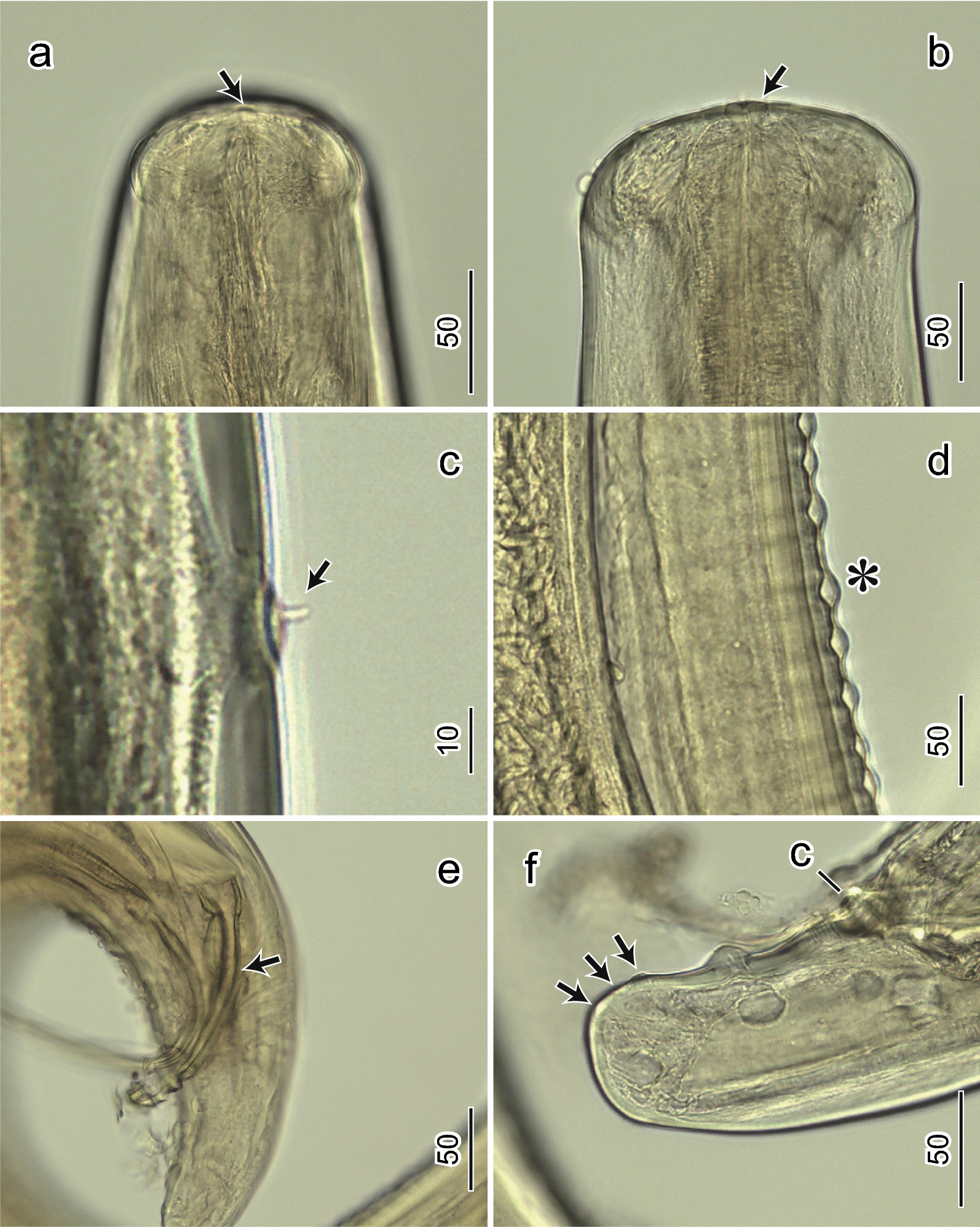
Light micrographs of *Deraiophoronema evansi*. Males (**a**–**f**). (**a**) Lateral view of the anterior end showing the pre-esophageal cuticular ring (arrow). (**b**) Dorsoventral view of anterior end showing pre-esophageal cuticular ring (arrow) and rectangular formation stretched laterally. (**c**) Deirid, lateral view (arrow). (**d**) Area rugosa with transverse bands (^*^), lateral view. (**e**) Right spicule, lateral view (arrow). (**f**) Lateral view of tail tip showing small terminal papillae (arrows) and cloaca (C). Scale bars are in micrometers

Male: (*n* = 2) (Table 3; Fig. 1j–p, 3a–f). Body, 98 (94–98) [96] mm long; width at mid-body, 240 (240–370) [305]. Muscular esophagus, 730 (730–750) [740] long. Glandular esophagus, 5.25 (5.25–6.25) [5.75] mm long. Total esophagus, 5.93 (5.93–7) [6.47] mm long. Nerve ring, 200 (200–212.5) [206.2] from the anterior end of body. Deirids, 312.5 (312.5–374.5) [343.3] from the anterior end of the body (Fig. 3c). Spicules unequal: right spicule shorter and thicker, 162.5 (162.5–175) [168.7] long (Figs. 1n, 3e), left spicule very elongated, 645 (645–700) [672.5] long. A total of eight pairs of large pedonculated papillae located at caudal region: four pre-cloacal and four post-cloacal papillae (Fig. 1p). Area rugosa beginning at level of first bend of tail, ending somewhat anterior to cloaca (Figs. 1m, p, 3d). Tail tapering with rounded tip, 100 (100–125) [112.5] long (Figs. 1p, 3f). No caudal alae, no gubernaculum, and no postdeirid. Measurements are in micrometers unless otherwise stated.

**Table 3:** Comparative morphometric data from *Deraiophoronema evansi* males and congeners recorded from camels. Measurements are in micrometers unless otherwise stated

Characteristics	Present study, range		Lewis 1882	Romanovitch 1916	Baylis et Daubney 1923	Boulenger 1924	Nagaty 1947	Sonin 1975^*^
Country	Egypt	Egypt	Pakistan	Kyrgyzstan	Pakistan	Pakistan	Egypt	Egypt
Examined material (no.)	Male (1)	Male (2)	Male	Male	Male	Male	Male	Male
Body length (mm)	98	94	76–114	70		75–90	80–110	
Body width	240	370	320–569	170		250–290	248–405	
Nerve ring from anterior end	200	213		176				190
Deirids from anterior end	313	374						
Muscular esophagus length	730	750				550	618–968	608–703
Glandular esophagus length (mm)	5.25	6.25					5.4–5.95	
Junction of esophagus/intestine (mm)	5.93	7						
Tail length	100	125	114	238		75–100	99–135	96–120
Right spicule	163	175	169		179	175–190	168–252	160–180
Left spicule	700	645	1000		1000	750–900	588–946	650–820
Pre-cloacal pairs	4	4				4	4	
Post-cloacal pairs	4	4				4	4	

Measurements are in micrometers unless otherwise stated

^*^Based on drawings from Nagaty 1947

Microfilaria: (*n* = 10). Body elongated, (256–270) [262] long. Width, (3.8–5.3) [4.6]. Anterior end rounded. Posterior end rounded. Sheathed (Figs. 1g–i, 2f). Measurements are in micrometers.

Remarks

*Deraiophoronema* Romanovitch 1916

Diagnosis: Setariinae Yorke and Maplestone, 1926. Anterior end with cuticular rectangular formation stretched laterally in both sexes. Body of uniform diameter throughout most of its length. Esophagus divided into anterior muscular and posterior glandular part. Deirid present. Caudal extremity of female with a round tail and a large terminal hump. Area rugosa precloacal, composed of transverse bands. Caudal papillae equally distributed before and after the cloaca. Spicules unequal, dissimilar. Right spicule shorter. Left spicule long and slender, divided into handle and lamina. Gubernaculum absent. Caudal alae absent. Postdeirid absent. Vulva anterior. Adult worms in tissues of its host mammals. Microfilariae with sheath in blood. Type species *Der. evansi* (Lewis, 1882), Romanovitch 1916.

### Phylogenetic analysis

**Fig. 4:**
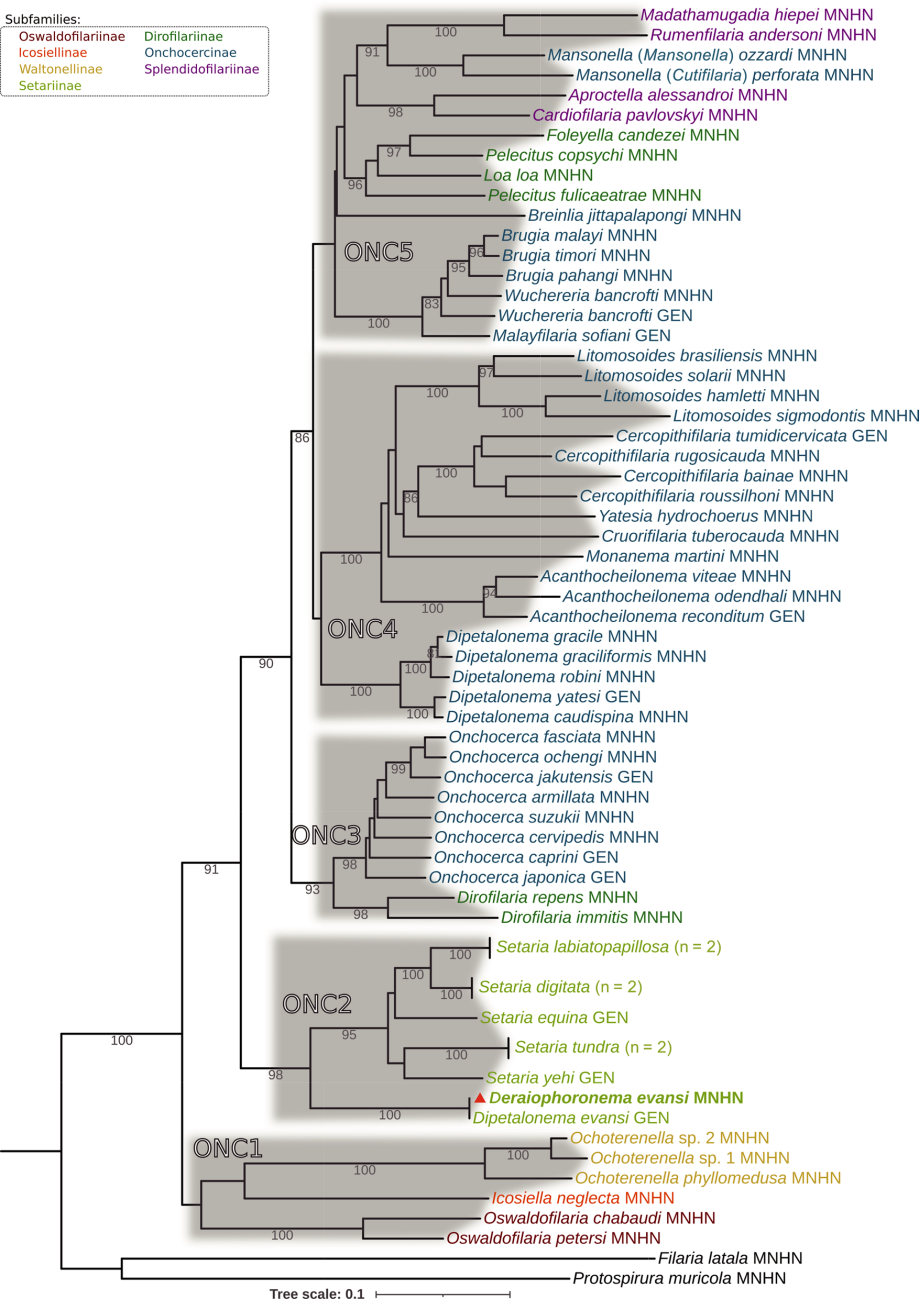
Filarial clades based on partitioned concatenated datasets of *12S* rDNA, *18S* rDNA, *28S* rDNA, *cox1*, *hsp70*, *myoHC*, and *rbp1* sequences using Bayesian inference (BI). The total length of the datasets is approximately 3690 bp. A total of 62 specimens of the Onchocercidae family (representing 58 species) were analyzed. *Filaria latala* and *Protospirura muricola* were used as outgroups. The topology was inferred using BI on two runs of 7 million generations, with the first 25% removed as burn-in. The weighted average of the best-fit evolution models in the GTR + I + G landscape was used for each gene. The onchocercid subfamilies present are linked to a color: Onchocercinae, blue; Dirofilariinae, dark green; Splendidofilariinae, purple; Setariinae, pale green; Waltonellinae, yellow; Icosiellinae, orange; and Oswaldofilariinae, red. Outgroups are represented in black. The red triangle indicates the sequences generated in this study. The scale bar below the diagram indicates the number of inferred changes along each branch. Sequences were analyzed at the Muséum National d’Histoire Naturelle (MNHN), Paris. Sequence data were obtained from GenBank (GEN)

Each ONC clade [31] is monophyletic (Fig. 4). The two *Der. evansi* taxa are consistently recovered as an early diverging lineage within the ONC2 clade, forming a strongly supported monophyletic sister group to the genus *Setaria*. The affiliations observed in the BI phylogeny were congruent with those from the ML analysis. All ONC clades, including ONC2, are robustly supported with a posterior probability (pp) of 1 (Additional File 2: Supplementary Fig. S1).

### *Wolbachia* detection

*Wolbachia* endosymbionts were not detected in the specimens.

## Discussion

On the basis of the morphological and molecular analyses, we ruled out *Acanthocheilonema* and *Dipetalonema* as valid genera for this species. First, a complete set of ten pairs of papillae is present on the tail in *Dipetalonema* and *Acanthocheilonema*, whereas current and previous morphological analysis of *Der. evansi* only revealed eight pairs (Fig. 1, Table 2). Second, the caudal extremity of both sexes is ornamented with conical processes, often called lappets in *Acanthocheilonema* or *Dipetalonema*, that have generic as well as specific value (three lappets in *Acanthocheilonema* or *Dipetalonema*), whereas current and previous morphological analysis of *Der. evansi* showed no lappets at the caudal extremity. Third, the anterior end with a cuticular rectangular formation stretched laterally in both sexes of *Der. evansi* indicated close affinities to the anterior end of *Setaria* species [37].

Regarding the phylogenetic relationships between *Der. evansi* and *Setaria* species, *Der. evansi* forms a distinct and well-supported monophyletic lineage that diverges early within the ONC2 clade, as sister group to *Setaria*. This phylogenetic position provides strong support for reassigning the genus *Deraiophoronema* from the subfamily Onchocercinae to the subfamily Setariinae (Fig. 4). In addition, *Wolbachia* endosymbionts were not detected in *Der. evansi* as in *Setaria* species [36, 38], whereas *Wolbachia* from the *Dipetalonema* species (*D. gracile*, *D. caudispina*, *D. robini*, and *D. graciliformis*) constitute a strongly supported clade, highlighting the validity of supergroup J [36, 39].

Before this study, Setariinae included three genera: *Setaria* Viborg, 1795 (with about 45 species), *Papillosetaria* Vevers, 1922 (with three species), and *Chabfilaria* Bain, Purnomo and Dedet, 1983 (with two species) [29]. Considering the new taxonomic position of *Der. evansi* and based on morphological characteristics presented herein, we ruled out the position of *Der. evansi* within the genera *Setaria*, *Papillosetaria*, and *Chabfilaria*. First, salient cephalic cuticular formations (circumoral elevations) are present in all *Setaria* species [37, 40] but not in *Der. evansi*. Second, a tail tip with conspicuous conical lappets, or with a large number of cuticular bosses/papillae is observed in *Setaria* and *Papillosetaria* [41], whereas there is none on the tail of *Der. evansi*. Third, a complete set of ten pairs of papillae and a gubernaculum are present in *Chabfilaria*, whereas only eight pairs of papillae and no gubernaculum are observed on *Der. evansi* [27].

Since its original description, the naming of *Deraiophoronema evansi* has been complex and confusing, mainly because the taxonomy of the genus *Dipetalonema* was unclear for a long time. Indeed, the genus *Dipetalonema *sensu stricto has been defined broadly by some authors and narrowly by others. In addition, the taxonomic history of the *Dipetalonema* lineage is also a challenge: numerous synonymies, elevation of subgenera to genera, and creation of new genera have strongly jammed the organization of this lineage. Altogether, it was difficult to relate what appeared to be well-defined genera to *Dipetalonema*, as synonyms were numerous.

The Dipetalonema lineage was first described in 1952 by Chabaud [23] with seven genera, i.e., *Ackertia*, *Breinlia*, *Skrjabinofilaria*, *Molinema*, *Acanthocheilonema*, *Loxodontofilaria*, and *Dipetalonema* [19]. Since then, only three, i.e., *Ackertia*, *Breinlia*, and *Skrjabinofilaria*, have not been taxonomically modified. The other six genera, i.e., *Macdonaldius*, *Monanema*, *Filarissima*, *Sprattia*, *Cercopithifilaria*, and *Yatesia*, have been integrated into the lineage and have not undergone any taxonomic changes [26, 42]. In 1976, *Molinema*, *Acanthocheilonema*, *Dipetalonema*, and *Loxodontofilaria* were lowered to subgenera of *Dipetalonema* [26]. In addition, the genus *Chenofilaria* was also lowered to a subgenus of *Dipetalonema* [26]. Finally, a new subgenus, *Orihelia*, was also added to include the species *D.* (*Orihelia*) *anticlava* in the *Dipetalonema* lineage [26]. Thus, at that time, the genus *Dipetalonema* included six subgenera. Later, in 1982, this taxonomic change was reversed by elevating *Molinema* and *Acanthocheilonema* to the rank of genus [42]. In addition, *Chenofilaria* was synonymized with *Acanthocheilonema* on the basis of the “very long divided esophagus and robust buccal capsule” of the type species *C. filaria*. The three other subgenera of the *Dipetalonema* lineage were maintained, and a fourth subgenus was added: *Dasypafilaria* [43]. Finally, the subgenus classification was abandoned in 2014 [29], and thus *Orihelia*, *Dasypafilaria*, and *Loxondotofilaria* were elevated to genera. The subgenus *Dipetalonema* also disappeared; hence, the genus *Dipetalonema* no longer has any subgenera attached to it. Currently, 14 genera would be in the *Dipetalonema* lineage on the basis of morphological characteristics: *Ackertia* (2 species), *Breinlia* (18 species), *Skrjabinofilaria* (monotypic), *Macdonaldius* (5 species), *Monanema* (5 species), *Filarissima* (monotypic), *Sprattia* (2 species), *Cercopithifilaria* (27 species), *Yatesia* (monotypic), *Molinema* (8 species), *Acanthocheilonema* (15 species), *Dipetalonema* (6 species), *Orihelia* (monotypic), and *Dasyfilaria* (monotypic).

Of these 14 genera, only 6 have at least one molecularly characterized species. The six sequenced genera are mainly phylogenetically positioned in the ONC4 clade, with the genus *Dipetalonema* sensu stricto positioned as a basal subclade in relation to the other taxa of the clade. The presence of the genus *Litomosoides*, which is morphologically related to but distinct from the *Dipetalonema* lineage, distally in ONC4, makes the lineage paraphyletic within ONC4. In addition, one of the genera of the lineage, *Breinlia*, is positioned in ONC5. The *Dipetalonema* lineage, if accurate, is therefore polyphyletic.

There are six species in the genus *Dipetalonema*, which parasitize Neotropical primates: *D. gracile* (Rudolphi, 1809), *D. caudispina* (Molin, 1858), *D. graciliformis* (Freitas, 1964), *D. robini* (Petit, Bain, Roussilhon, 1985), *D. freitasi* (Bain, Diagne, Muller, 1987), and *D. yatesi* (Notarnicola, Jiménez, Gardner, 2007). The genus is clearly monophyletic and apart from the ONC4 (Fig. 4).

Regarding the other genera used to describe *Der. evansi* in the past, *Acanthocheilonema* are parasites of carnivores, insectivores, pinnipeds, and rodents (Holarctic and Afrotropical realms), and *Setaria* are parasites of ungulates and Hyracoidea (worldwide). In addition, for the Setariinae, *Papillosetaria* are parasites of edentates (Neotropical realm), *Chabfilaria* are parasites of edentates (Neotropical Realm), and *Deraiophoronema* are parasites of camelids (Paleartic, Afrotropical, and Indomalayan realms, introduced in Australia).

## Conclusions

We analyzed *Dipetalonema evansi* in camels and reassessed its taxonomy and evolutionary position in the Onchocercidae. This species is reassigned to *Deraiophoronema evansi* (Lewis, 1882) Romanovitch 1916 n. comb. (syn: *Dipetalonema evansi*). Taking into account both morphological features and genetic divergence, we conclude that *Der. evansi* is most closely related to the genus *Setaria*. On the basis of morphological features, we have ruled out the possibility of this species belonging to all three genera, *Setaria*, *Papillosetaria*, and *Chabfilaria*. We validated the genus *Deraiphoronema* and placed it as the fourth genus of the Setariinae. We did not detect *Wolbachia* in *Der. evansi*, confirming the absence of endosymbiont bacteria in clades ONC1 and ONC2. Clarifying the taxonomy of *Der. evansi* enhances our understanding of camelid filarial parasites and may support accurate diagnosis, epidemiological surveillance, and targeted control strategies in regions where camel filariosis is endemic.

## Supplementary Information

Additional file 1.

Additional file 2. Figure S1. Filarial clades based on partitioned concatenated datasets of *12S* rDNA, *18S* rDNA, *28S* rDNA, *cox1*, *hsp70*, *myoHC, *and* rbp1 *sequences using maximum likelihood (ML) inference. The total length of the datasets is approximately 3690 bp. 62 specimens of the Onchocercidae family (representing 58 species) were analysed. *Filaria latala *and *Protospirura muricola *were used as outgroups. The best-fitting substitution model was determined using the corrected version of the Akaike Information Criterion (AICc). The topology was inferred using 1000 bootstraps. The onchocercid subfamilies present are linked to a colour: Onchocercinae: blue, Dirofilariinae: dark green, Splendidofilariinae: purple, Setariinae: pale green, Waltonellinae: yellow, Icosiellinae: orange, and Oswaldofilariinae: red.

## Data Availability

The data supporting the conclusions of this article are included within the article and its additional files. The neotypes of *Deraiophoronema evansi* and the paratypes were deposited in the MNHN, Paris, France, under accession nos. MNHN-IN-110YT and MNHN-IN-111YT, respectively. Sequences were deposited in the GenBank database under the accession numbers provided in Additional File 1: Supplementary Table S1. .

## Abbreviations

n. comb.: *Combinatio nova* [new combination]
MNHN: Muséum National d’Histoire Naturelle
ca.: Approximately
syn.: Synonym
*cox1*: Cytochrome C oxidase subunit I
rDNA: Ribosomal DNA
*MyoHC*: Myosin heavy chain
*rbp1*: RNA polymerase II large subunit
*hsp70*: 70 Kilodalton heat shock proteins
ML: Maximum likelihood
*ftsZ*: Filamenting temperature-sensitive mutant Z
*dnaA*: Chromosomal replication initiator protein
*fbpA*: Fructose-bisphosphate aldolase
*gatB*: Aspartyl/glutamyl-tRNA(Asn/Gln) amidotransferase subunit B
PCR: Polymerase chain reaction
