# Supplementary figures and images for: Morphological redescription and taxonomic reassignment of Deraiophoronema evansi (Lewis, 1882) Romanovitch 1916 n. comb. (syn: Dipetalonema evansi) (Spirurida: Onchocercidae) from camels
Source: Parasit Vectors. 2025 Oct 10;18:406. doi: 10.1186/s13071-025-07019-z (PMC12512819; doi:10.1186/s13071-025-07019-z)

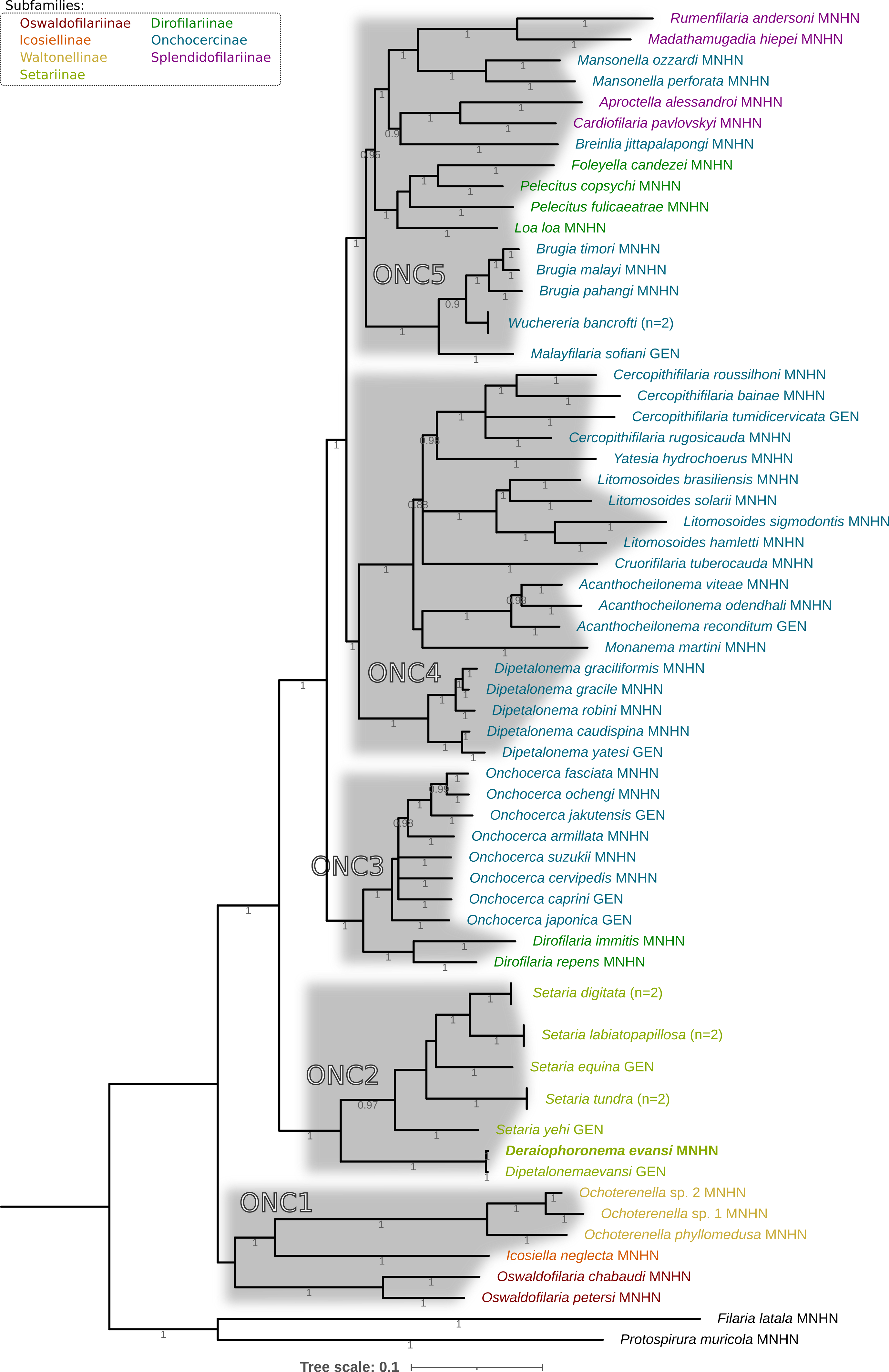

Supplement: Supplementary file 2 — Additional file 2. Figure S1. Filarial clades based on partitioned concatenated datasets of 12S rDNA, 18S rDNA, 28S rDNA, cox1, hsp70, myoHC, and rbp1 sequences using maximum likelihood (ML) inference. The total length of the datasets is approximately 3690 bp. 62 specimens of the Onchocercidae family (representing 58 species) were analysed. Filaria latala and Protospirura muricola were used as outgroups. The best-fitting substitution model was determined using the corrected version of the Akaike Information Criterion (AICc). The topology was inferred using 1000 bootstraps. The onchocercid subfamilies present are linked to a colour: Onchocercinae: blue, Dirofilariinae: dark green, Splendidofilariinae: purple, Setariinae: pale green, Waltonellinae: yellow, Icosiellinae: orange, and Oswaldofilariinae: red. [file 13071_2025_7019_MOESM2_ESM.png]
